# The Utility of the SYNTAX Score II and SYNTAX Score 2020 for Identifying Patients with Three-Vessel Disease Eligible for Percutaneous Coronary Intervention in the Multivessel TALENT Trial: A Prospective Pilot Experience

**DOI:** 10.31083/j.rcm2304133

**Published:** 2022-04-08

**Authors:** Kai Ninomiya, Patrick W. Serruys, Scot Garg, Hironori Hara, Shinichiro Masuda, Shigetaka Kageyama, Nozomi Kotoku, Emelyne Sevestre, Abhishek Kumar, Peter O’Kane, Azfar Zaman, Bruno Farah, Michael Magro, Rohit M. Oemrawsingh, Helge Möllmann, Nicolas Meneveau, Stephan Achenbach, Julien Lemoine, Abdelhakim Allali, Sean Gallagher, Joanna Wykrzykowska, Maciej Lesiak, Marc Silvestri, William Wijns, Faisal Sharif, Yoshinobu Onuma

**Affiliations:** ^1^Department of Cardiology, National University of Ireland, Galway (NUIG), H91 TK33 Galway, Ireland; ^2^Department of Cardiology, Academic Medical Center, University of Amsterdam, 1105 AZ Amsterdam, The Netherlands; ^3^Department of Cardiology, Royal Blackburn Hospital, BB2 3HH Blackburn, UK; ^4^Department of Cardiology, Wrightington, Wigan and Leigh NHS Foundation Trust, WN1 2NN Wigan, UK; ^5^Department of Cardiology, Royal Bournemouth Hospital, BH7 7DW Bournemouth, UK; ^6^Department of Cardiology, Freeman Hospital, NE7 7DN Newcastle, UK; ^7^Department of Interventional Cardiology, Clinique Pasteur, 31300 Toulouse, France; ^8^Department of Cardiology, Elisabeth-TweeSteden Ziekenhuis, 5042AD Tilburg, The Netherlands; ^9^Department of Cardiology, Albert Schweitzer Hospital, 3318 AT Dordrecht, The Netherlands; ^10^Department of Cardiology, St. Johannes Hospital, 44137 Dortmund, Germany; ^11^Department of Cardiology, University Hospital Jean Minjoz, 25000 Besançon, France; ^12^Department of Cardiology, Friedrich-Alexander University Erlangen-Nürnberg, 91054 Erlangen, Germany; ^13^Department of Cardiology, Clinique Louis Pasteur, 54270 Nancy, France; ^14^Department of Cardiology, Heart Center, Segeberger Kliniken, 23795 Bad Segeberg, Germany; ^15^Department of Cardiology, University Hospital of Wales, CF14 4XW Cardiff, UK; ^16^Department of Cardiology, University Medical Center Groningen, 9713 GZ Groningen, The Netherlands; ^17^Department of Cardiology, Poznan University of Medical Sciences, 61-701 Poznan, Poland; ^18^Department of Cardiology, GCS ES-Axium-Rambot, 13090 Aix-en-Provence, France

**Keywords:** coronary artery bypass grafts (CABG), percutaneous coronary intervention (PCI), risk stratification, SYNTAX score

## Abstract

**Background:**

Personalized prognosis plays a vital role in deciding between percutaneous coronary intervention (PCI) and coronary artery bypass grafting (CABG) in patients with three-vessel disease (3VD). The aim of this study is to compare the modality of revascularization chosen by the local heart team to that recommended by using individualized predictions of medium, and long-term all-cause mortality amongst patients with 3VD screened in the Multivessel TALENT trial.

**Methods:**

The SYNTAX score II (SS-II) and SS-2020 were evaluated in 200 consecutive patients by a core laboratory and compared to the decision of the “on site” heart team.

**Results:**

According to the SS-II, CABG was the recommended treatment in 51 patients (25.5%) however 34 (66.6%) of them received PCI. According to SS-2020 the predicted absolute risk differences (ARD) between PCI and CABG were significantly higher in patients receiving CABG compared to those treated by PCI for major adverse cardiovascular and cerebrovascular events, a composite of all-cause mortality, stroke or myocardial infarction at 5-years (8.8 ± 4.6% vs 6.0 ± 4.0%, *p *< 0.001) and all-cause mortality at 5- (5.2 ± 3.5% vs 3.7 ± 3.0%, *p* = 0.008) and 10-years (9.3 ± 4.8% vs 6.2 ± 4.2%, *p *< 0.001). Based on the novel threshold of equipoise (individual absolute risk differences [ARD] <4.5%), 133 patients were eligible for PCI however 23 of them underwent CABG; conversely, amongst the 67 patients where CABG was recommendation (individual ARD >4.5%), only 19 received it.

**Conclusions:**

Despite the robustness of the risk models proposed for screening, several deviations from the recommended mode of revascularization were observed by the core laboratory among the first 200 patients with 3VD screened in the Multivessel TALENT trial.

**Clinical Trial Registration:**

ClinicalTrials.gov reference: NCT04390672.

## 1. Introduction

Selecting the optimal modality of revascularization in patients with three-vessel disease (3VD) and/or left main coronary artery disease (LMCAD) remains a topic of debate for patients, and between non-invasive cardiologists, interventional cardiologists and cardiac surgeons [1]. Ultimately the decision between percutaneous coronary intervention (PCI) and coronary artery bypass graft (CABG) surgery should be made by consensus during a heart team consultation as endorsed by a Class I, Level C recommendation from the European Society of Cardiology (ESC) [2].

In 2009 the anatomical SYNTAX score (aSS) was incorporated into the ESC and American College of Cardiology (ACC) guidelines for revascularization, and subsequently PCI was endorsed as an alternative to CABG in patients with aSS <23; however, in 2019 diabetes mellitus became an additional limitation for selecting PCI in patients with 3VD, with or without LMCAD, thereby downgrading the previous recommendation from Level IA to IIb [2].

In 2013 the aSS was combined with relevant clinical characteristics and comorbidities and renamed the SYNTAX score II (SS-II), and subsequent prospective testing in the EXCEL trial showed it to predict global (mixed, surgical and percutaneous) all-cause mortality at 4-years, however it failed to correctly predict survival in each arm of the trial [3].

Recently the SS-II has been redeveloped and recalibrated after integrating very long-term all-cause mortality from the SYNTAX trial (SYNTAXES trial; NCT03417050) [4]. The new score—SYNTAX score 2020 (SS-2020)—has additional capability for predicting all-cause mortality [5, 6], and has been externally validated not only in other randomized trials with long-term follow-up of patients with 3VD, with or without LMCAD [6], but also in a contemporary registry [7].

The present study is a comparison between the modality of revascularization chosen at site by the local heart team, and the recommendation based on using the SS-II and SS-2020, as calculated by the core laboratory (CL) during their screening of patients with 3VD to determine their eligibility for PCI, and subsequent enrolment in the ongoing Multivessel TALENT trial, which will compare clinical outcomes in patients randomised to treatment with drug eluting coronary stents with thin or ultra-thin struts.

## 2. Methods

### 2.1 Study Population

The Multivessel TALENT trial (NCT04390672) is an ongoing randomized trial comparing the use of the SUPRAFLEX Cruz (Sahajanand Medical Technologies, Mumbai, India) sirolimus-eluting stent and the SYNERGY (Boston Scientific, Natick, MA, USA) everolimus-eluting stent in patients with *de novo* 3VD without LMCAD [8].

The trial incorporates all the components of “best practice” which were previously implemented and tested in the SYNTAX II trial [9]; namely, prospective selection of patients eligible for percutaneous treatment of 3VD through the use of the SS-II score for predicting all-cause mortality at 4 years after either PCI or CABG [5]. Only patients with a recommendation for PCI or with equipoise between PCI and CABG are eligible for enrolment. In addition, Quantitative Flow Ratio (QFR) is assessed to determine which lesion(s) can be deferred, and which hemodynamically significant lesions need to be treated (functional SYNTAX score). Post stent implantation, the adequacy of stent treatment must be assessed by QFR post-PCI, and intravascular ultrasound (IVUS) or optical coherence tomography (OCT) to ensure optimal stent apposition and deployment, as well as complete lesion coverage. Moreover, the recently validated strategy of monotherapy with P2Y12 inhibitors after 1 month of dual antiplatelet therapy (DAPT) is the recommended antiplatelet regimen instead of conventional DAPT [2, 10].

The present study includes the first 200 consecutive patients screened for the trial that have been reviewed by a Data Safety Monitoring Board (DSMB) at the time of their first predefined evaluation [8]. The study complied with the Declaration of Helsinki and Good Clinical Practice. Provisional 5 years follow up is part of the patient informed consent.

### 2.2 The SYNTAX Score Family 

Currently, a web-based and smartphone application facilitate the computation of the various SYNTAX scores (https://syntaxscore2020.com/).

The aSS assesses the complexity and extent of coronary disease according to a weighting score, related to the amount of subtended myocardium at risk [11]. Additional scoring points related to the complexity of the anatomy (e.g., bifurcation, calcium, and tortuosity…) are incorporated into the score [12].

The aSS is converted into a functional SYNTAX score (fSS) by a central CL (CORRIB Core Lab, Galway, Ireland) following physiological assessment using QFR of each stenotic lesion visually detected on cine fluoroscopy [13]. Anatomic scoring points are subtracted if the stenotic vessel is not physiologically significant as indicated by a QFR >0.8 (**Supplementary Fig. 1**).

This anatomical/functional SYNTAX score has been merged with clinical characteristics and comorbidities using a logistic regression formula, which includes two anatomical and 11 clinical prognostic factors [14], to predict 2-year mortality in all-comers populations treated exclusively with PCI.

These probabilistic formulas for predicting major cardiac and cerebrovascular events (MACCE) and all-cause mortality have been expanded with the development of the SS-II and SS-2020. The SS-II can be used in patients with 3VD and LMCAD randomized to CABG or PCI, and uses two anatomical effect modifiers (the aSS and the presence of 3VD or LMCAD) and 6 clinical prognostic factors (age, sex, chronic obstructive pulmonary disease [COPD], peripheral vascular disease [PVD], creatinine clearance, and left ventricular ejection fraction [LVEF]) to predict 4-year all-cause mortality [5]. In summary, the SS-II affords a personalized recommendation between: (i) CABG only; (ii) PCI only; or (iii) equipoise of PCI and CABG. In the present study the SS-II was calculated by investigators and presented to the local heart team in order to evaluate the patient’s eligibility for PCI prior to their informed consent and randomization.

The SS-2020 was redeveloped from the 10-year follow-up of the SYNTAXES trial and externally validated in four randomized trials (FREEDOM, BEST, PRECOMBAT, and EXCEL) and a large contemporary registry of patients with 3VD with or without LMCAD treated with PCI or CABG [7, 15]. The score, which uses two anatomical effect modifiers (the aSS and the presence of 3VD or LMCAD) and 7 clinical prognostic factors (age, medically treated diabetes mellitus with or without insulin, COPD, PVD, current smoking, creatinine clearance, and LVEF), predicts 5-year MACCE defined as all-cause mortality, stroke, or myocardial infarction, and 5- and 10- year all-cause mortality [6, 15].

### 2.3 Angiography Derived Physiology (Quantitative Flow Ratio, QFR)

Following written informed consent but prior to randomization, QFR was analyzed off-line with the QAngio XA 3D/QFR imaging software (Medis Medical Imaging Systems, Leiden, The Netherlands) in the CL (CORRIB Core Lab, Galway, Ireland), by analysts unaware of the patient’s baseline characteristics [7]. The anatomical and functional SYNTAX scores were also evaluated by the CL and slides showing the individual colour coded QFR analyses of each stenotic vessel were subsequently provided to the investigators (**Supplementary Fig. 1**).

### 2.4 Statistical Analysis 

Continuous variables were expressed as mean ± standard deviation and were compared using Student’s *t*-test or Mann–Whitney U test. Categorical variables were reported as numbers and percentages and were compared using the Chi square or Fisher’s exact test as appropriate. A two-sided *p*-value < 0.05 was considered statistically significant.

The predicted individual absolute risk differences (ARD) in all-cause mortality between CABG and PCI for each patient were ranked in order of magnitude according to the predicted PCI mortality minus the predicted CABG mortality and shown in a scatter plot of predicted mortality with either PCI or CABG (Fig. 2). The dots in the scatter plot were connected with the use of locally estimated scatterplot smoothing (LOESS) curves. The external validation of the SS-2020 in the CREDO-Kyoto registry has shown that an individual predicted ARD in all-cause death at 5-year of <4.5% and ≥4.5% offers a sensible cut-off for “equipoise of PCI and CABG” or “CABG better”, respectively [7].

The level of agreement between the “on site” heart team treatment and the corelab recommendation based on the SS-II and SS-2020 was assessed by Cohen’s kappa. Patients whose treatment on site was PCI, and whose recommendation based on the SS-II and SS-2020 was “PCI only” or “equipoise of PCI and CABG”, were considered concordant.

Analyses were performed using SPSS Statistics, version 26 (IBM Corp., Armonk, NY, USA), Stata 15 (Stata Corp, College Station, TX, USA) and R version 3.6.0 (R Foundation for Statistical Computing, Vienna, Austria).

## 3. Results 

**Fig. 1.**
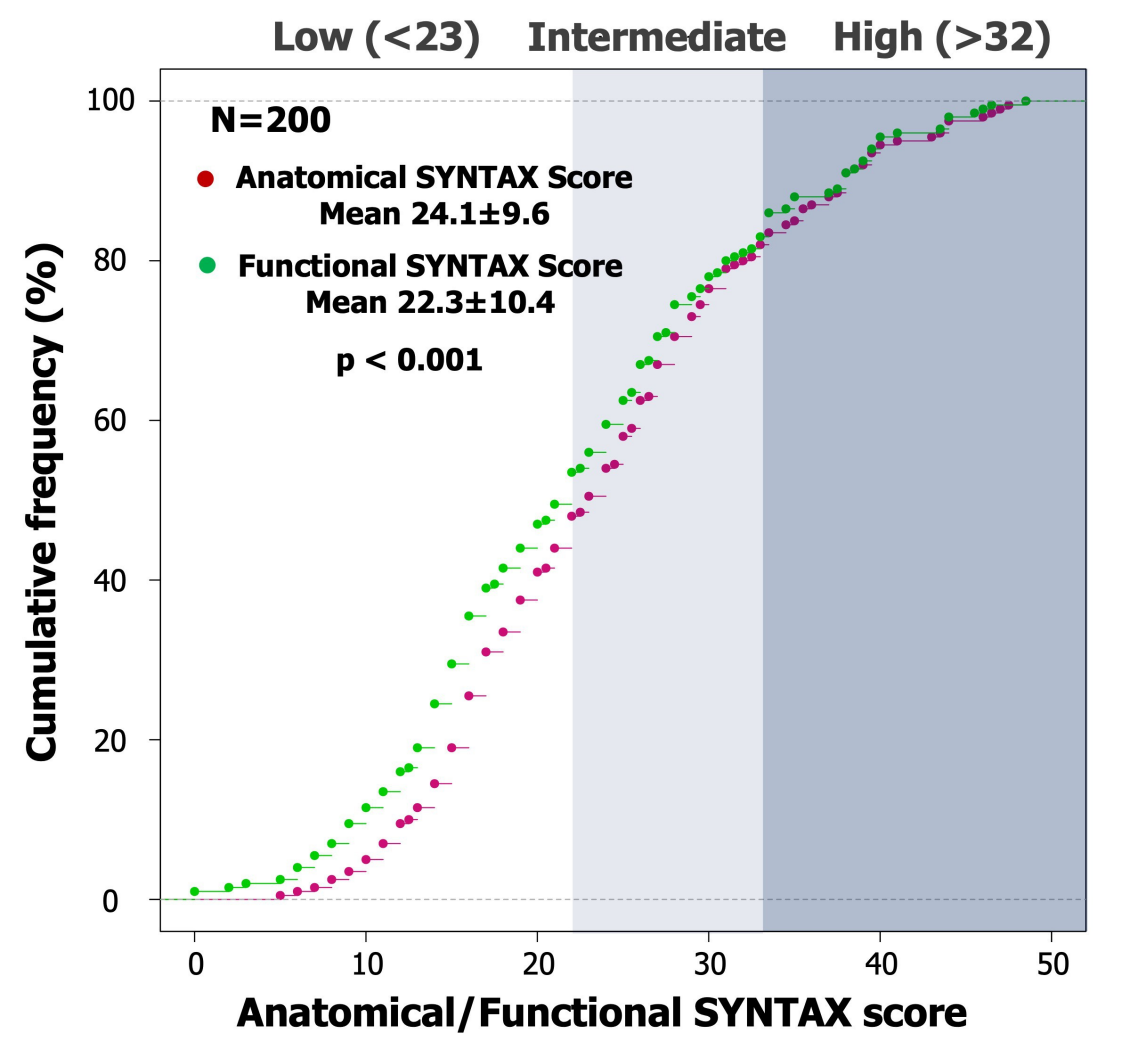
**Cumulative frequency curve of anatomical and functional SYNTAX score**. Based on QFR functional assessment, the cumulative frequency curve of the anatomical SYNTAX score after functional adjustment showed a significant leftwards shift with a significant reduction of the median value (24.1 ± 9.6 vs 22.3 ± 10.4, *p <* 0.001).

Among 200 consecutive patients with 3VD, 158 and 42 patients respectively received PCI and CABG, following discussion at the local heart team. Patient characteristics are shown in Table 1. As expected, patients treated with CABG versus PCI had higher anatomic and functional SYNTAX scores. Of the 200 patients, 40 (20%) had a high aSS (>33), 72 (36%) an intermediate aSS (>22 and <33), and 88 (44%) a low aSS score (<22). In 191 patients (95.5%), QFR values were measured in all 3 major coronary arteries and using this QFR functional assessment the cumulative frequency curve of the aSS after functional adjustment showed a significant leftwards shift, with a significant reduction in the median value from 24.1 ± 9.6 to 22.3 ± 10.4 (*p *< 0.001, Table 1, Fig. 1).

**Table 1.** **Baseline characteristics for the SYNTAX score family**.

Variables used in the SYNTAX score family		Overall patients (n = 200)	Patients treated with PCI (n = 158)	Patients treated with CABG (n = 42)	*p* value
Age		68.0 ± 9.1	68.3 ± 9.0	66.7 ± 9.8	0.314
Male		81.0 (162)	78.5 (124)	90.5 (38)	0.119
Body mass index (kg/$m^{2}$)		28.5 ± 5.3	28.7 ± 5.5	27.9 ± 4.6	0.385
Diabetes		31.0 (62)	32.3 (51)	26.2 (11)	0.574
	Medically treated diabetes	28.5 (57)	29.1 (46)	26.2 (11)	0.848
	Insulin	8.5 (17)	10.1 (16)	2.4 (1)	0.131
Creatinine clearance (mL/min)		77.3 ± 24.1	77.2 ± 25.8	77.8 ± 16.6	0.886
LVEF (%)		54.7 ± 9.0	54.4 ± 9.2	55.9 ± 8.2	0.351
COPD		10 (20)	10.1 (16)	9.5 (4)	1.000
PVD		9.0 (18)	9.5 (15)	7.1 (3)	0.770
Previous stroke		6.0 (12)	6.3 (10)	4.8 (2)	1.000
Current smoker		21.0 (42)	21.5 (34)	19.0 (8)	0.833
Hemoglobin (g/dL)		13.9 ± 1.8	13.8 ± 1.8	14.1 ± 1.6	0.469
WBC (${\mathrm{10}}^{9}$ cells/L) *		7.6 ± 1.9	7.7 ± 1.9	7.1 ± 2.2	0.097
Anatomical SYNTAX score		24.1 ± 9.6	21.7 ± 8.4	33.0 ± 8.4	<0.001
Functional SYNTAX score		22.3 ± 10.4	20.2 ± 9.2	30.5 ± 10.4	<0.001

Data are presented as mean ± standard deviation, or percentage (number). CABG, coronary artery bypass grafting; COPD, chronic obstructive pulmonary disease; LVEF, left ventricular ejection fraction; PCI, percutaneous coronary intervention; PVD, peripheral vascular disease; WBC, white blood cell. *The value of WBC are missing in 5 patients.

Table 2 shows the predicted event rates derived from all the SYNTAX scores.

**Table 2.** **Predicted event rates**.

Predicted event rates	Overall patients (n = 200)	Patients treated with PCI (n = 158)	Patients treated with CABG (n = 42)	*p* value
4-year mortality after PCI (%)	12.1 ± 10.4	11.9 ± 10.8	12.8 ± 8.9	0.614
4-year mortality after CABG (%)	9.5 ± 10.1	9.4 ± 10.1	9.8 ± 10.1	0.830
4-year mortality ARD (%)	2.6 ± 8.2	2.5 ± 8.5	3.0 ± 7.3	0.709
5-year mortality after PCI (%)	16.8 ± 13.4	17.1 ± 13.9	15.7 ± 11.8	0.552
5-year mortality after CABG (%)	12.8 ± 11.1	13.4 ± 11.6	10.6 ± 8.7	0.140
5-year mortality ARD (%)	4.0 ± 3.2	3.7 ± 3.0	5.2 ± 3.5	0.008
5-year MACE after PCI (%)	23.8 ± 13.3	23.8 ± 13.6	23.7 ± 12.4	0.982
5-year MACE after CABG (%)	17.2 ± 10.5	17.8 ± 10.9	14.9 ± 8.7	0.122
5-year MACE ARD (%)	6.6 ± 4.3	6.0 ± 4.0	8.8 ± 4.6	<0.001
10-year mortality after PCI (%)	33.1 ± 21.1	33.5 ± 21.5	31.7 ± 19.7	0.623
10-year mortality after CABG (%)	26.3 ± 18.6	27.3 ± 19.1	22.4 ± 16.1	0.135
10-year mortality ARD (%)	6.9 ± 4.5	6.2 ± 4.2	9.3 ± 4.8	<0.001

Data are presented as mean ± standard deviation. ARD, absolute risk difference (PCI rate – CABG rate); CABG, coronary artery bypass grafting; MACE, major cardiac and cerebrovascular events; PCI, percutaneous coronary intervention.

According to SS-II and SS-2020, the predicted ARDs for MACCE and all-cause mortality at 5- and 10-years were significantly higher in patients receiving CABG by the heart team, compared to those enrolled into the PCI Multivessel TALENT trial (predicted ARD for 5-year MACE, 5-year and 10-year mortality if receiving to PCI vs CABG; 8.8 ± 4.6% vs 6.0 ± 4.0%, *p *< 0.001, 5.2 ± 3.5% vs 3.7 ± 3.0%, *p* = 0.008, 9.3 ± 4.8% vs 6.2 ± 4.2%, *p *< 0.001, respectively; Table 2). Of note, the ARD for mortality in the overall population, which can be considered a surrogate for the average treatment effect, increased with the duration of follow up, from 2.6% at 4 years, to 4.0% at 5 years and 6.9% at 10 years.

According to the four-year all-cause mortality predicted by the SS-II, 51 (25.5%) of the 200 screened patients should have received CABG, however two-thirds of them (n = 34) underwent PCI (Table 3). Paradoxically, 25 of the 146 patients who had predicted equipoise in mortality were referred for surgery. The level of agreement between the treatment recommended using the SS-II and the “on site” heart team was slight (Cohen’s kappa = 0.18, 95% confidence interval [CI] 0.00–0.35), with a concordance in treatment selection of 70.5%.

**Table 3.** **Treatment decision and recommendation based on the SYNTAX score II**.

		Treatment recommendation		
		Eligible for PCI	Recommended CABG	Total
Actual treatment	PCI	124	34	158
	CABG	25	17	42
	Total	149	51	200
Cohen’s kappa = 0.18 (95% CI 0.00–0.35)				

CI, confidence interval; CABG, coronary artery bypass grafting; PCI, percutaneous coronary intervention.

**Fig. 2.**
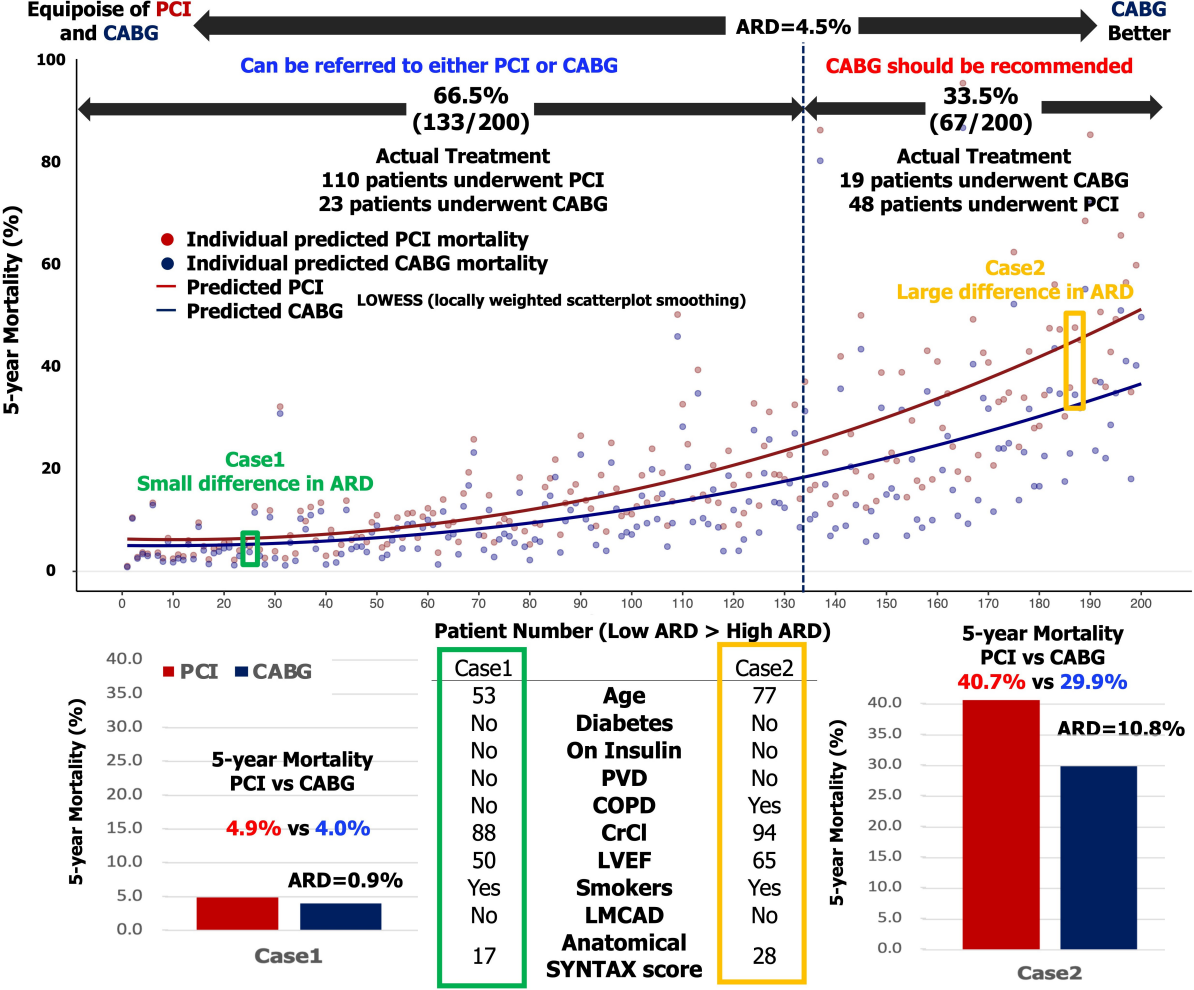
**Treatment recommendation according to the predicted absolute risk difference for 5-year mortality**.

External validation of the SS-2020 in the contemporary cohort of the CREDO-Kyoto registry, which used new generation drug-eluting stents and mandated peri-procedural intra-vascular imaging, has established that an individual predicted ARD in all-cause death at 5-year of <4.5% and ≥4.5% offers a sensible cut-off for “equipoise of PCI and CABG” or “CABG better”, respectively [7]. According to this criterion, CABG would have been appropriate in 67 patients (individual ARD >4.5%) of whom 19 actually received it, whereas PCI or CABG could have been equally selected in 133 patients (individual ARD <4.5%), of whom 23 received surgery (Table 4 and Fig. 2). The level of agreement between the treatment recommended using the SS-2020 and the “on site” heart team treatment was also slight (Cohen’s kappa = 0.12, 95% CI –0.04–0.29), with a concordance in treatment selection of 64.5%.

**Table 4.** **Treatment decision and recommendation according to the SYNTAX score 2020 accepting a predicted absolute risk difference in mortality at 5-year with PCI of less than 4.5% as threshold criterion (the Credo Kyoto criterion) for a legitimate PCI from a long-term survival perspective**.

		Treatment recommendation		
		Eligible for PCI	Recommended CABG	Total
Actual treatment	PCI	110	48	158
	CABG	23	19	42
	Total	133	67	200
Cohen’s kappa = 0.12 (95% CI –0.04–0.29)				

Recommendation is based on the absolute risk difference (ARD) of the predicted 5-year mortality rates. If ARD is <4.5%, recommendation is eligible for PCI. If ARD is >4.5%, recommendation is CABG. CI, confidence interval; CABG, coronary artery bypass grafting; PCI, percutaneous coronary intervention.

(Upper) Predicted mortality after either PCI (red dots) or CABG (blue dots) for each individual patient (individual scatterplots). The dots in the scatter plot were connected with the use of locally estimated scatterplot smoothing (LOESS) curves. (Bottom) In case 1, the patient has predicted 5-year mortality rates of 4.9% after PCI and 4.0% after CABG. The predicted ARD is 0.9% (<4.5%); in the external validation of SYNTAX 2020 in the Credo-Kyoto registry, patients with an ARD <4.5% showed equipoise in mortality on Kaplan Meier estimates therefore, the patient can be referred for either PCI or CABG. In case 2, the patient has predicted 5-year mortality rates of 40.7% after PCI and 29.9% after CABG. The predicted ARD is 10.8% (>4.5%), hence CABG should be recommended. In whole population, CABG would have been appropriate in 67 patients of whom 19 received it, whereas either PCI or CABG could have been equally selected in 133 patients of whom 23 received CABG.

## 4. Discussion

The main findings of this study are as follows:

(1) Among the first 200 consecutive patients with 3VD without LMCAD screened in the Multivessel TALENT trial, 158 patients underwent PCI, whereas 42 underwent CABG.

(2) Despite the ability to calculate prognostic scores using a website and/or smartphone application, several deviations from the mode of revascularization recommended by the SS-II and SS-2020 were observed by the central CL during this preliminary tele medicine experience of multi-center screening.

(3) Only accurate personalized predictions of vital prognosis, which have been validated by very long term follow up of randomized trials and registries, will ultimately convince practitioners and scientific societies that personalized treatment recommendations should be rigorously implemented.

Among the first 200 consecutive patients with 3VD but without LMCAD screened for the Multivessel TALENT trial, 158 underwent PCI and 42 CABG. If we were to apply the CREDO-KYOTO threshold criterion for legitimate PCI (i.e., an ARD for mortality <4.5%) to the current cohort then the percentage of legitimate PCI would be 66.5% (Table 4 and Fig. 2).

The SS-II was derived from medium-term follow-up of the SYNTAX trial using Cox proportional hazards, and interactions of the anatomic SYNTAX score with clinical characteristics of patients randomized to PCI or CABG in the SYNTAX trial. It only predicts all-cause mortality at 4 years and is only applicable to a randomized population with 3VD, with or without LMCAD. Notably, it is also the only score that has so far been prospectively tested, in the EXCEL trial [3]. The SS-2020 is derived from 10-year follow-up of the SYNTAXES study, and uses calibration plots to predict 5-year MACCE and 5- and 10-year all-cause death. The individual vital prognosis provided by these two scores could help the heart team make an appropriate decision between percutaneous and surgical revascularization, with the objective information provided by the scores possibly helping the surgeon, interventional cardiologist, non-invasive cardiologist, and patient accept the decision more readily.

### 4.1 Legitimacy of Including Three Vessel Disease in a Trial Comparing Two Different Stents 

In a PCI trial involving a population with 3VD, it is vital to legitimize percutaneous treatment and ensure compliance with the ESC guidelines for revascularization and functional testing. Theoretically, only non-diabetic patients with an anatomic SYNTAX score <23 are eligible for PCI according to recommendations from the ESC [2]. Moreover, the operator has the obligation, even in the presence of an ischemic non-invasive test, to identify in the epicardial vessels and their branches the flow limiting lesions that must be treated, and conversely the stenotic lesions for which treatment can be deferred [2].

Guidelines rely on evidence-based medicine derived from past trials, whilst trialists try to envision new ways to practice medicine and test new concepts. In the Multivessel TALENT trial, as in the SYNTAX II trial, patient eligibility for PCI was determined not only by coronary anatomy (anatomic SYNTAX score <23) but also by using the anatomical, functional and clinical criteria embodied by the SS-II [5]. Of note, the SS-2020 was published shortly after the publication of the Multivessel TALENT design [8].

The legitimate choice of PCI as the modality of revascularization, as decided by a multidisciplinary heart team is not actually part of the Multivessel TALENT trial, however it is mandated by the ESC/ACC guidelines, as a pre-requisite step prior to specific informed consent related to the trial’s inclusion and exclusion criteria. Having selected appropriate and legitimate candidates for PCI, the central core lab provides investigators with a three vessel assessment of angiography derived physiology (QFR), to be used as a tool for pre-procedural identification of those flow limiting lesions that need to be treated.

The central CL, on-site investigators and heart team all had access to the same software application used for computation (https://syntaxscore2020.com/) of the various scores during screening of the first 200 PCI candidates in the trial. However, despite this there was a substantial discordance between the mode of revascularization as recommended by the scores calculated by the investigators, and by the CL. This deviation from evidence-based treatment recommendations requires critical appraisal since it is a possible cause of concern in this pilot experience of screening for PCI eligibility. Notably in the randomized cohorts of the EXCEL trial, deviation from the treatment recommended by the score (i.e., PCI instead of CABG) due to the imposed randomized trial allocation led to an excess of death [16]. An uncontrolled observational study from a PCI center without on-site surgery in Serbia has previously reported similar findings [17].

### 4.2 Can We Rely on the Heart Team Decision? 

Detailed recommendations for implementing a heart team are not provided in the current guidelines, potentially limiting their utilization in clinical practice and leading to reduced quality of care. In contemporary practice the Heart team discussion continues to be a vital part of the decision-making process for patients with complex coronary disease and retains a Class IA recommendation (C level, without randomized approach) [3], despite the encouraging report of the first virtual attempt to randomize heart teams in the SYNTAX III trial [18].

Recently, Ma *et al*. [19] have reported the agreement between heart teams for revascularization decisions in patients with complex coronary artery disease and the potential factors behind discrepancies. Despite the fact that the Heart teams were in possession of the key factors for the selecting the mode of revascularization (e.g., SYNTAX score, STS score etc…), the primary outcome kappa for the level of agreement for inter-team decision-making was moderate (kappa = 0.58), at variance with the randomized SYNTAX III trial in which the kappa for agreement for inter-team decision-making based on Invasive Conventional Cine Angiography (ICA) or Computerized Tomographic Angiography (CTA) was “almost perfect” (kappa = 0.82) [18].

In the present study, the heart team consultation was not standardized, although the use of the SS-II was recommended and facilitated by electronic media provided by the academic sponsor (https://syntaxscore2020.com/). Nevertheless, only a slight agreement was seen between the treatment recommendations according to the scores and the actual treatment (kappa = 0.18) with a concordance of treatment selection according to the SS-II of 70.5%.

### 4.3 Current Actuality of the SYNTAX Score 2020 (SS-2020)

Improvements in devices and the techniques for stent implantation with intravascular imaging guidance, combined with better antiplatelet regimens and secondary prevention have reduced all-cause mortality following PCI over the last 10-years. In the SYNTAX II trial, there was a significant reduction in 5-year all-cause mortality compared to the SYNTAX I PCI cohort (8.1% vs 13.8%, *p* = 0.013) [20]. Similarly, all-cause mortality following CABG fell from 8.5% in the SYNTAX trial to 5.5% in the more contemporary EXCEL trial. Recently in FAME 3 trial, a randomized trial with 3VD and similar patient baseline characteristics as in the SYNTAX trial, all-cause mortality at 1 year in the FFR-guided PCI arm was 2.8% lower than in the SYNTAX trial (4.4% vs 1.6%) [21]. This is probably the reason why the threshold of equipoise in ARD for mortality moved to 4.5% in the external validation of CREDO KYOTO cohort [6]; below that threshold criterion the Kaplan Meier estimates show equipoise in mortality. Today, the use of an individual predicted ARD >0% with the SS-2020, derived from outdated technology and techniques, seems to be too restrictive since it leads to the recommendation of CABG in almost all patients with 3VD without LMCAD.

### 4.4 Patient’s Information and Perspective 

From a patient’s perspective, PCI is less invasive and this remains a very attractive and persuasive factor in favour of PCI, even though the individual predicted fatal outcome based on objective evidence, may formally contradict the patient’s preference; of note, probabilistic outcome predictions are seldom shared with patients [22].

Therefore, it is mandatory to use validated models of personalized prediction on long term vital prognosis when deciding between modalities of revascularization. It is even more critical when patients with 3VD, who are potentially candidates for CABG, are enrolled in a PCI trial testing new stents. The eligibility of these patients for percutaneous treatment must be discussed in advance and agreed with surgeons and patients.

Only accurate personalized predictions of vital prognosis, validated by observed all-cause mortality from very long-term follow up of randomized trials, will ultimately convince practitioners and scientific societies that personalized treatment recommendations should be rigorously implemented.

## 5. Limitation 

The present study investigates predicted event rates based solely on pre-procedural angiographic anatomy and physiology, as well as clinical characteristics. However, operator proficiency, technical improvements in devices and the impact of novel pharmacological strategies may subsequently modulate the accuracy of these predictions based on preprocedural determinants [23].

Equipoise in all-cause mortality, though an unbiased end point for trialists, is not ultimately the most relevant measure of a treatment’s benefit from a patient’s perspective, and quality adjusted life year (QALY) of survival remains the ultimate goal in a holistic conception of medicine [24].

## 6. Conclusions 

Among the first 200 consecutive patients with 3VD, screened on site in the Multivessel TALENT trial, 158 patients underwent PCI, while 42 recieved CABG following discussions at the local heart team. Notably, several of these treatment decisions were at variance with the personalized treatment recommendations provided by validated individual prognostic scores [14]. Scientific endorsement, the logistics of implementation, regulatory enforcement and further prospective evaluation are the challenges of future decision-making scores, which should be openly shared with patients.

## Supplementary Material

Supplementary material associated with this article can be found, in the online version, at https://doi.org/10.31083/j.rcm2304133.

## References

[1] Serruys PW, Ono M, Garg S, Hara H, Kawashima H, Pompilio G (2021). Percutaneous Coronary Revascularization: JACC Historical Breakthroughs in Perspective. *Journal of the American College of Cardiology*.

[2] Neumann FJ, Sousa-Uva M, Ahlsson A, Alfonso F, Banning AP, Benedetto U (2019). 2018 ESC/EACTS Guidelines on myocardial revascularization. *European Heart Journal*.

[3] Campos CM, van Klaveren D, Farooq V, Simonton CA, Kappetein A, Sabik JF (2015). Long-term forecasting and comparison of mortality in the Evaluation of the Xience Everolimus Eluting Stent vs. Coronary Artery Bypass Surgery for Effectiveness of Left Main Revascularization (EXCEL) trial: prospective validation of the SYNTAX Score II. *European Heart Journal*.

[4] Serruys PW, Morice M, Kappetein AP, Colombo A, Holmes DR, Mack MJ (2009). Percutaneous Coronary Intervention versus Coronary-Artery Bypass Grafting for Severe Coronary Artery Disease. *New England Journal of Medicine*.

[5] Farooq V, van Klaveren D, Steyerberg EW, Meliga E, Vergouwe Y, Chieffo A (2013). Anatomical and clinical characteristics to guide decision making between coronary artery bypass surgery and percutaneous coronary intervention for individual patients: development and validation of SYNTAX score II. *The Lancet*.

[6] Takahashi K, Serruys PW, Fuster V, Farkouh ME, Spertus JA, Cohen DJ (2020). Redevelopment and validation of the SYNTAX score II to individualise decision making between percutaneous and surgical revascularisation in patients with complex coronary artery disease: secondary analysis of the multicentre randomised controlled SYNTAXES trial with external cohort validation. *The Lancet*.

[7] Hara H, Shiomi H, van Klaveren D, Kent DM, Steyerberg EW, Garg S (2021). External Validation of the SYNTAX Score II 2020. *Journal of the American College of Cardiology*.

[8] Hara H, Gao C, Kogame N, Ono M, Kawashima H, Wang R (2020). A randomised controlled trial of the sirolimus-eluting biodegradable polymer ultra-thin Supraflex stent versus the everolimus-eluting biodegradable polymer SYNERGY stent for three-vessel coronary artery disease: rationale and design of the Multivessel TALENT trial. *EuroIntervention*.

[9] Glineur D, Wijns W (2019). The 2010-2014-2018 trilogy of ESC-EACTS Guidelines on myocardial revascularisation: we cannot jump three steps this way and then return to where we began. *EuroIntervention*.

[10] Watanabe H, Domei T, Morimoto T, Natsuaki M, Shiomi H, Toyota T (2019). Effect of 1-Month Dual Antiplatelet Therapy Followed by Clopidogrel vs 12-Month Dual Antiplatelet Therapy on Cardiovascular and Bleeding Events in Patients Receiving PCI: The STOPDAPT-2 Randomized Clinical Trial. *Journal of the American Medical Association*.

[11] Sianos G, Morel MA, Kappetein AP, Morice MC, Colombo A, Dawkins K (2005). The SYNTAX Score: an angiographic tool grading the complexity of coronary artery disease. *EuroIntervention*.

[12] Serruys P, Onuma Y, Garg S, Sarno G, van den Brand M, Kappetein A (2009). Assessment of the SYNTAX score in the Syntax study. *EuroIntervention*.

[13] Nam C, Mangiacapra F, Entjes R, Chung I, Sels J, Tonino PAL (2011). Functional SYNTAX score for risk assessment in multivessel coronary artery disease. *Journal of the American College of Cardiology*.

[14] Chichareon P, van Klaveren D, Modolo R, Kogame N, Takahashi K, Chang CC (2021). Predicting 2-year all-cause mortality after contemporary PCI: Updating the logistic clinical SYNTAX score. *Catheterization and Cardiovascular Interventions*.

[15] Takahashi K, van Klaveren D, Steyerberg EW, Onuma Y, Serruys PW (2021). Concerns with the new SYNTAX score – Authors’ reply. *The Lancet*.

[16] Modolo R, Chichareon P, van Klaveren D, Dressler O, Zhang Y, Sabik JF (2020). Impact of non-respect of SYNTAX score II recommendation for surgery in patients with left main coronary artery disease treated by percutaneous coronary intervention: an EXCEL substudy. *European Journal of Cardio-thoracic Surgery*.

[17] Stanetic BM, Ostojic M, Campos CM, Marinkovic J, Farooq V, Kovacevic-Preradovic T (2017). ApPropriateness of myocaRdial RevascularizatiOn assessed by the SYNTAX score II in a coUntry without cardiac Surgery faciliTies; PROUST study. *International Journal of Cardiology*.

[18] Collet C, Onuma Y, Andreini D, Sonck J, Pompilio G, Mushtaq S (2018). Coronary computed tomography angiography for heart team decision-making in multivessel coronary artery disease. *European Heart Journal*.

[19] Ma HP, Lin S, Li X, Dou KF, Yang WX, Feng W (2021). Exploring optimal heart team protocol to improve decision-making stability for complex coronary artery disease: a sequential explanatory mixed method study. *European Heart Journal - Quality of Care and Clinical Outcomes*.

[20] Banning AP, Serruys P, De Maria GL, Ryan N, Walsh S, Gonzalo N (2021). Five-year outcomes after state-of-the-art percutaneous coronary revascularization in patients with de novo three-vessel disease: final results of the SYNTAX II study. *European Heart Journal*.

[21] Fearon WF, Zimmermann FM, De Bruyne B, Piroth Z, van Straten AHM, Szekely L (2022). Fractional Flow Reserve–Guided PCI as Compared with Coronary Bypass Surgery. *New England Journal of Medicine*.

[22] Taggart DP (2005). Surgery is the best intervention for severe coronary artery disease. *British Medical Journal*.

[23] Kawashima H, Serruys PW, Ono M, Hara H, O’Leary N, Mack MJ (2021). Impact of Optimal Medical Therapy on 10-Year Mortality After Coronary Revascularization. *Journal of the American College of Cardiology*.

[24] Abdallah MS, Wang K, Magnuson EA, Osnabrugge RL, Kappetein AP, Morice M (2017). Quality of Life after Surgery or DES in Patients with 3-Vessel or Left Main Disease. *Journal of the American College of Cardiology*.

