# Supplementary material for: The Utility of the SYNTAX Score II and SYNTAX Score 2020 for Identifying Patients with Three-Vessel Disease Eligible for Percutaneous Coronary Intervention in the Multivessel TALENT Trial: A Prospective Pilot Experience
Source: Rev Cardiovasc Med. 2022 Apr 8;23(4):133. doi: 10.31083/j.rcm2304133 (PMC11273643; doi:10.31083/j.rcm2304133)
Supplement: Supplementary file 1 [file 2153-8174-23-4-133-s1.zip › Supplementary Material.docx]

**Supplementary Material**

**Supplementary Fig. 1. QFR results in screened patients.** (S1a) Case 1. The anatomic SYNTAX score was 39.5. Vessel QFRs of right coronary artery (RCA) and left anterior descending artery (LAD) were 0.64 and 0.47, respectively. There was a total occlusion of left circumflex artery (LCX). According to the results of QFR, the functional SYNTAX score was the same as the anatomic SYNTAX score (39.5). Anatomic syntax points for each vessel are shown in the figures highlighted in yellow. Although the patient had an anatomic SYNTAX score greater than 22, the patient underwent percutaneous coronary intervention (PCI). (S1b) Case 2. The anatomic SYNTAX score was 15. Vessel QFRs of RCA, LAD, and LCX were 0.62, 0.51, and 0.90, respectively. According to the results of QFR, the functional SYNTAX score was calculated as 12 since the lesion in the circumflex did not need to be revascularized. The patient could have been treated by PCI, but actually the three vessels were bypass grafted (CABG).


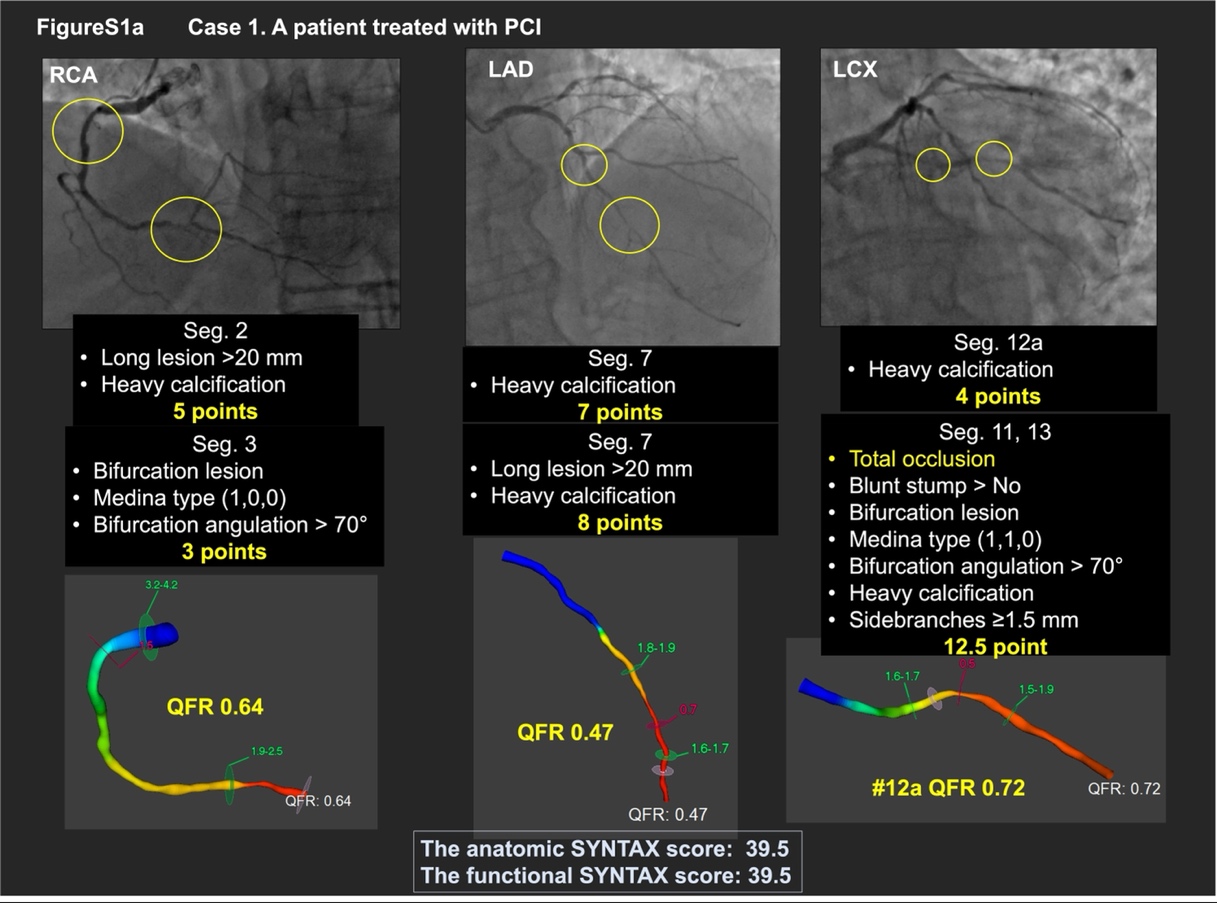

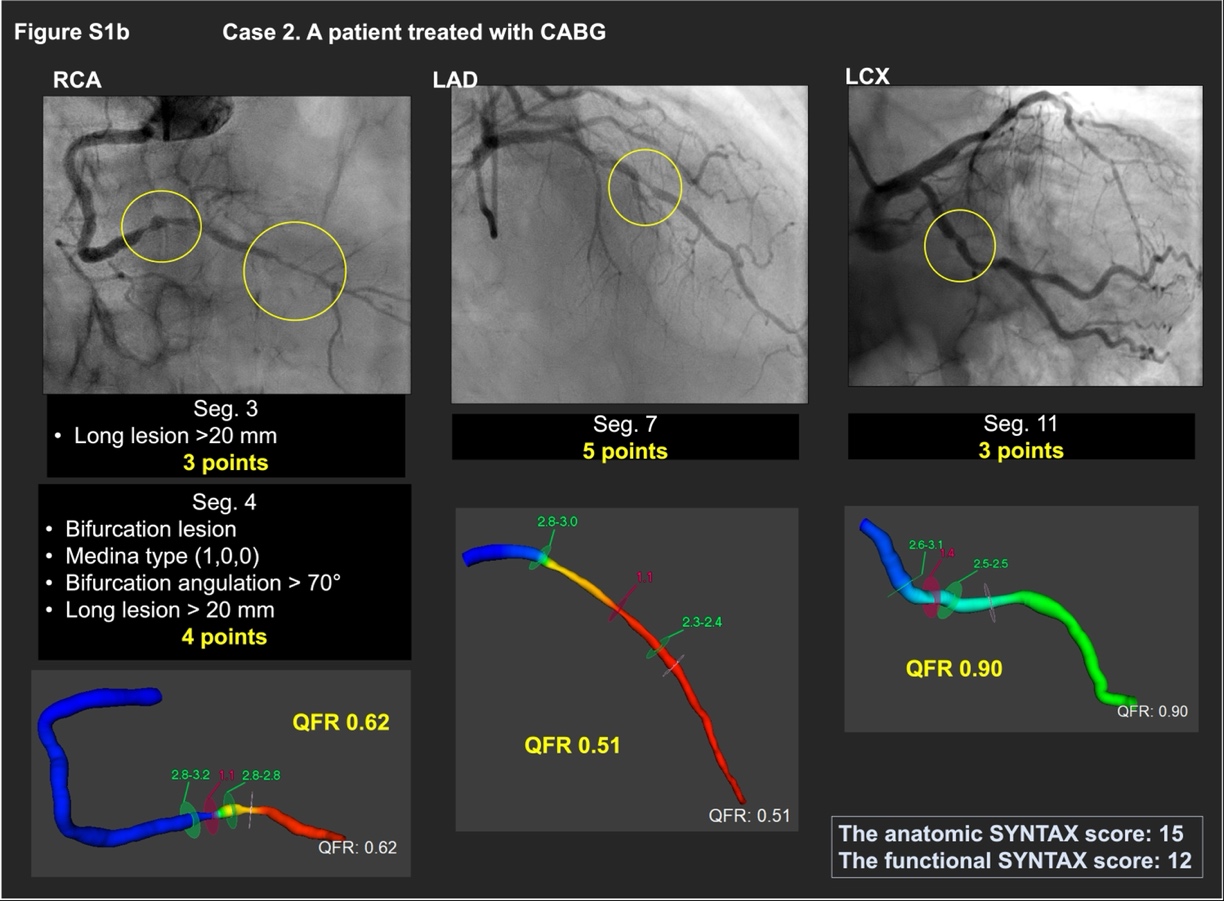


## Reproducibility of anatomical SYNTAX Score (aSS)

The aSS is a major component of the SS-II and SS-2020, however, its calculation is subject to intra-and inter-observer variability. Serruys *et al*. [1] reported weighted kappa values for inter- and intra-observer reproducibility of the aSS of 0.45 and 0.59, respectively; notably the aSS as calculated by investigators consistently underscored the corelab by 3.4 points. Subsequently, Généreux *et al* [2] have demonstrated that highly reproducible SYNTAX score measurements (Fleiss’ Kappa = 0.84) can be achieved by experienced CL technicians and agreement among interventional cardiologist can be improved (Fleiss’ Kappa = 0.76) after further training with the CL. We have to concede that objective and automated assessment by machine learning of fluoroscopic or CTA would promote fast, accurate and reproducible aSS [3]. In the current study the aSS was calculated by both sites and the CL, with the mean aSS higher when assessed by the CL than site investigators (24.1 ± 9.6 vs 22.5 ± 10.2, *p* = 0.018, Supplementary Figure2). However, the agreement of the proportion of patients classified in aSS tertiles (low <23, intermediate 23–32, and high >32) was almost perfect (weighted Kappa = 0.85) (Supplementary Table1). Nevertheless, the level of agreement between the treatment recommendation based on SS-II and SS-2020 and the "on site" heart team treatment were slight.

**
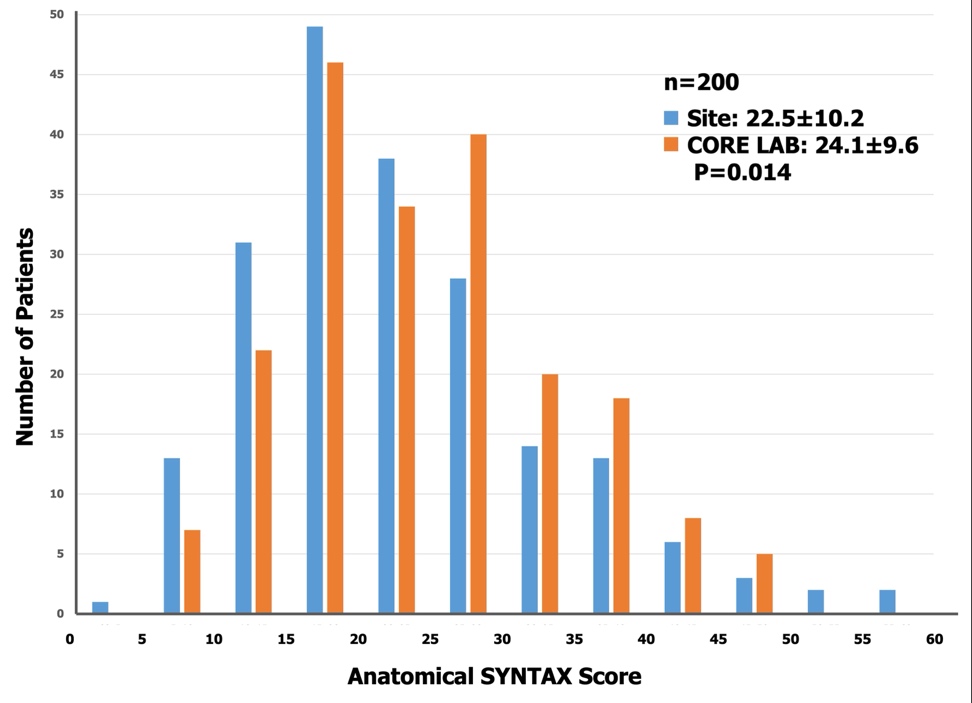
**

**Supplementary Fig. 2. Anatomical SYNTAX Score; Site vs Core laboratory.** In 151 out of 200 patients, Anatomical SYNTAX Score (aSS) was calculated by both sites and core laboratory. The mean aSS was higher when assessed by CL than by site investigators (24.1 ± 9.6 vs 22.5 ± 10.2, *p* = 0.014).

Supplementary Table 1. Comparison of risk stratification by sites and core laboratory.

| **Anatomical SYNTAX Score (aSS) assessed by Site vs Core laboratory (CL)** | | | | | |
| --- | --- | --- | --- | --- | --- |
|  |  | CL aSS | | |  |
|  |  | Low | Intermidiate | High | Total |
| Site aSS | Low (<23) | 80 (40.0%) | 25 (12.5%) | 10 (5.0%) | 115 |
|  | Intermidiate | 13 (6.5%) | 28 (14.0%) | 14 (7.0%) | 55 |
|  | High (>32) | 4 (2.0%) | 10 (5.0%) | 16 (8.0%) | 30 |
|  |  | 97 | 63 | 40 | 200 |
| **Weighted Kappa = 0.85** | | | | | |
|  |  |  |  |  |  |

The agreement of the proportion of patients classified in Anatomical SYNTAX score tertiles (low <23, intermediate 23–32, and high >32) was almost perfect (weighted Kappa = 0.85).

References:

[1] Serruys P, Onuma Y, Garg S, Sarno G, van den Brand M, Kappetein A, *et al*. Assessment of the SYNTAX score in the Syntax study. EuroIntervention. 2009; 5: 50–56.

[2] Généreux P, Palmerini T, Caixeta A, Cristea E, Mehran R, Sanchez R, *et al*. SYNTAX Score Reproducibility and Variability between Interventional Cardiologists, Core Laboratory Technicians, and Quantitative Coronary Measurements. Circulation: Cardiovascular Interventions. 2011; 4: 553–561.

[3] Serruys PW, Chichareon P, Modolo R, Leaman DM, Reiber JHC, Emanuelsson H, *et al*. The SYNTAX score on its way out or … towards artificial intelligence: part I. EuroIntervention. 2020; 16: 44–59.
