# Supplementary figures and images for: The Utility of the SYNTAX Score II and SYNTAX Score 2020 for Identifying Patients with Three-Vessel Disease Eligible for Percutaneous Coronary Intervention in the Multivessel TALENT Trial: A Prospective Pilot Experience
Source: Rev Cardiovasc Med. 2022 Apr 8;23(4):133. doi: 10.31083/j.rcm2304133 (PMC11273643; doi:10.31083/j.rcm2304133)

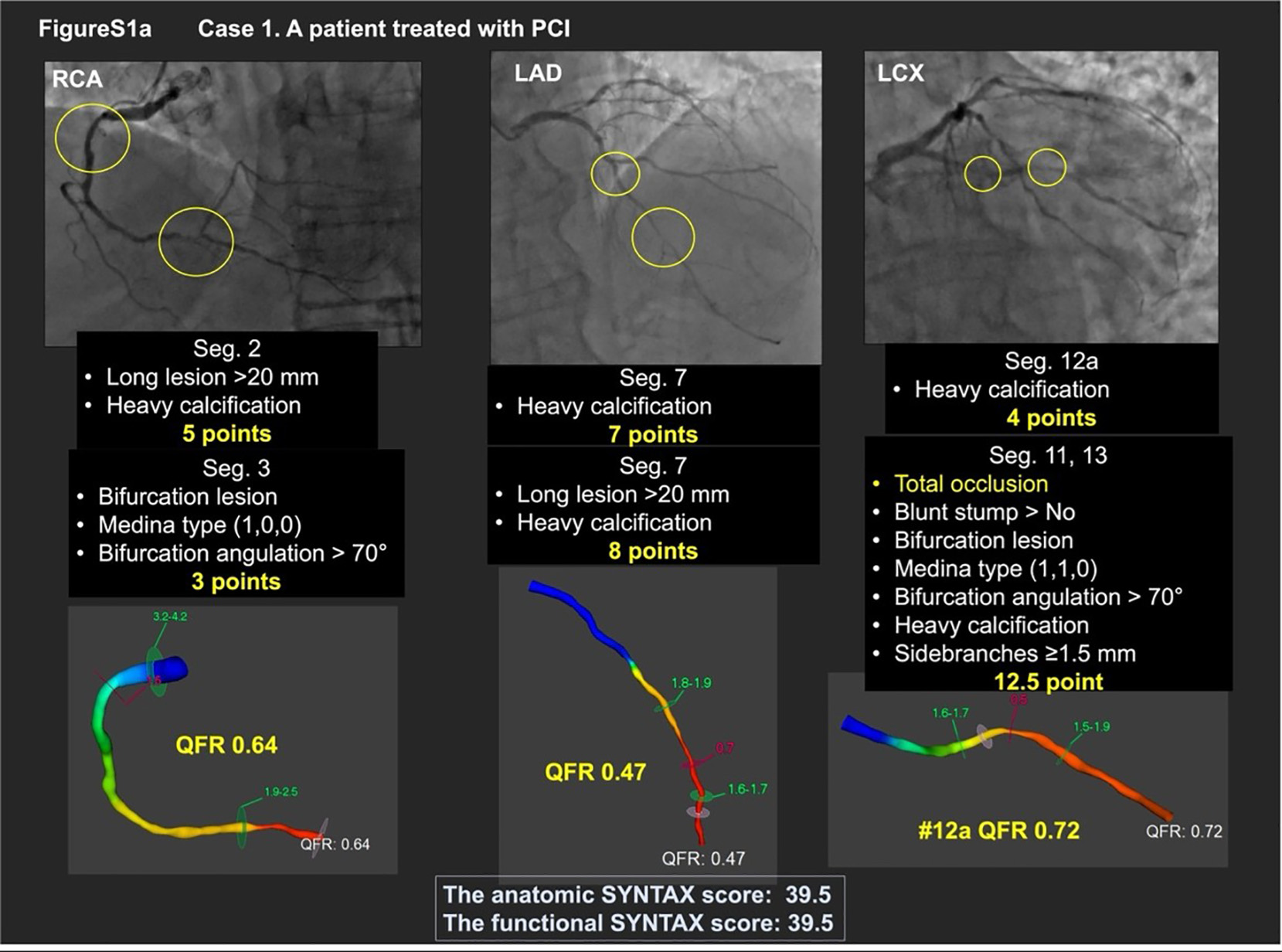

Supplement: Supplementary file 1 [file 2153-8174-23-4-133-s1.zip › Supplementary Fig. 1a.jpg]

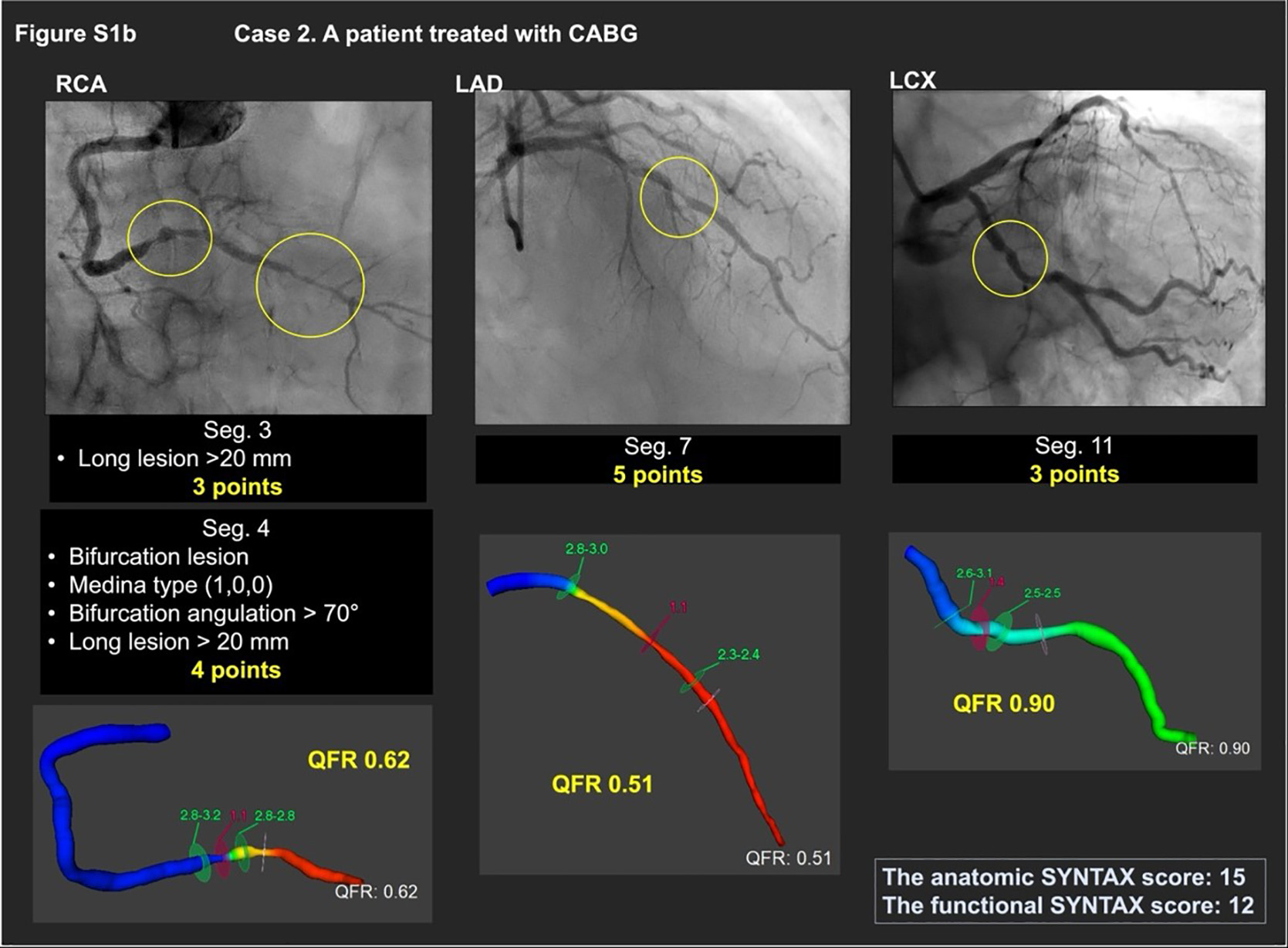

Supplement: Supplementary file 1 [file 2153-8174-23-4-133-s1.zip › Supplementary Fig. 1b.jpg]

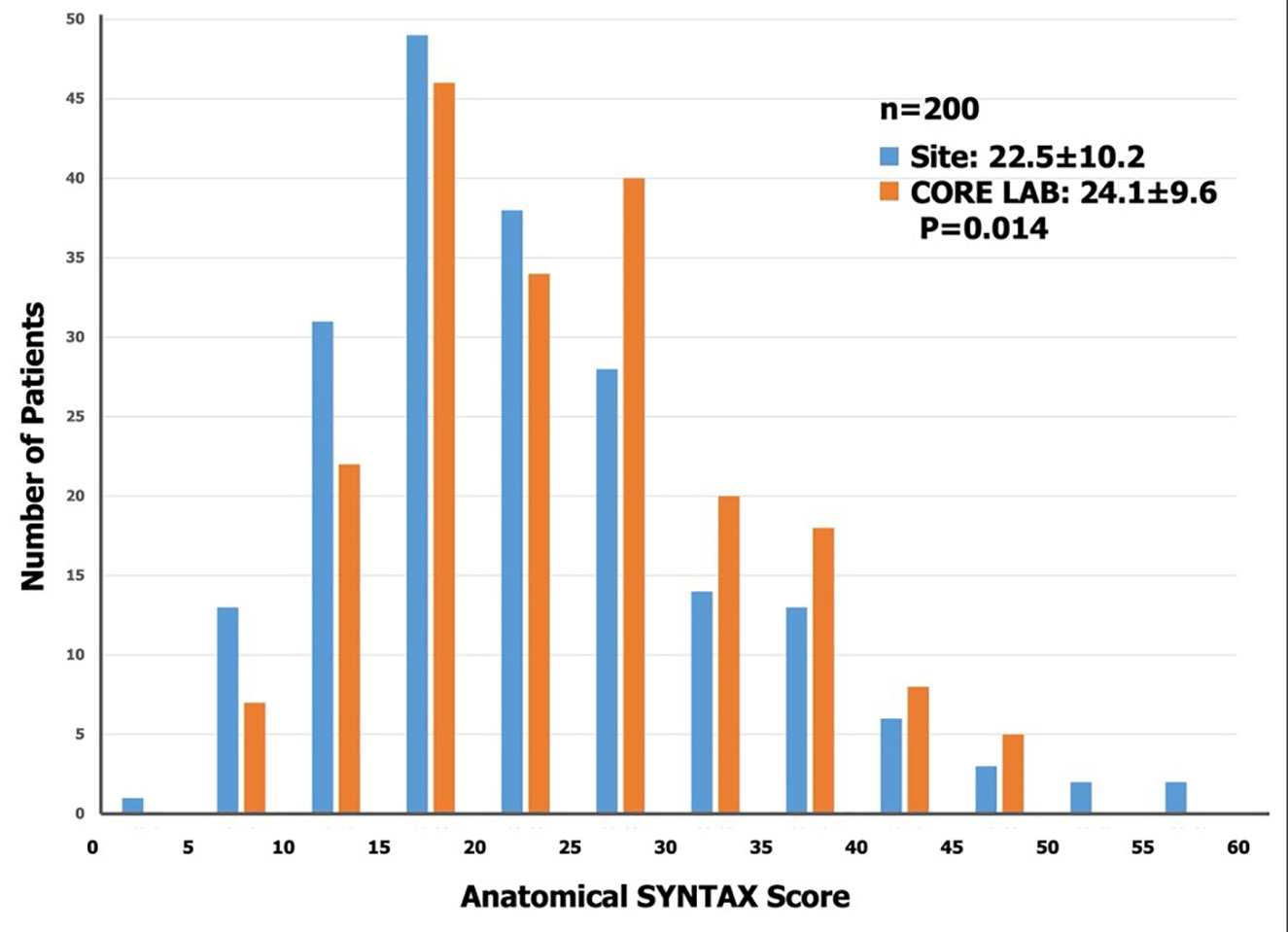

Supplement: Supplementary file 1 [file 2153-8174-23-4-133-s1.zip › Supplementary Fig. 2.jpg]
